# Substance Use and Anxiety About Pain Among Patients Seeking Abortion Services

**DOI:** 10.7759/cureus.57034

**Published:** 2024-03-27

**Authors:** Megan Masten, Jeanelle Sheeder, Aaron Lazorwitz

**Affiliations:** 1 Obstetrics and Gynecology, Complex Family Planning, University of Colorado School of Medicine, Aurora, USA; 2 Obstetrics and Gynecology, University of Colorado School of Medicine, Aurora, USA; 3 Divisions of Family Planning and Reproductive Sciences, Yale School of Medicine, New Haven, USA

**Keywords:** opioid, cannabidiol, marijuana, anxiety, pain, abortion, substance use

## Abstract

Objectives: To evaluate how recent opioid, marijuana, and cannabidiol use affects pre-procedure pain-related anxiety for patients seeking abortion.

Methods: We conducted a prospective, cross-sectional anonymous survey of patients seeking abortion assessing recent substance use and anxiety about pain during and after abortion. We compared substance users' and non-users' anxiety scores.

Results: Among 217 participants, recent opioid users (5.3%) had higher median anxiety scores for pain during (7.0 vs 6.0; p=0.33) and after (8.0 vs 6.0; p=0.01) abortion than non-opioid users. Anxiety scores were similar for marijuana and cannabidiol users.

Conclusions: Assessing recent opioid use may help guide counseling for anxiety about abortion.

## Introduction

Roughly one in four women in the United States (US) will have an elective termination of pregnancy by the age of 45 with the majority (88%) of these abortions occurring in the first trimester [1]. Substance use in the US is also increasingly common. Based on the 2019 National Survey of Drug Use and Health, 21.3% of women ages 18-25 and 8.1% of women ages 26 and older had used marijuana in the last month [2]. Further, 4.6 million women ages 12 and older misused opioids in the past year [2]. Opioid pain prescription misuse in Colorado is estimated at 4.6% based on data from 2015-2019, which is slightly higher than the national average (3.7%) [2].

Although both pregnancy termination and substance use are common, a paucity of published literature exists regarding people seeking pregnancy termination who use substances, especially women with opioid use disorder [3]. Research has suggested that fear of pain and catastrophizing can increase pain intensity ratings [4]. Emerging research suggests that pain perception may also be heightened for individuals using substances such as marijuana, cannabidiol (CBD), or opioids [5]. The currently published literature on substance use and abortion has focused on pain control during abortion procedures for this patient population but has yet to evaluate opioid users without a formal diagnosis [6]. 

A knowledge gap in the literature surrounds how recent use of substances affects anxiety or worry about abortion-related pain. To gather preliminary data on this knowledge gap, we utilized an anonymous survey to evaluate how recent use of marijuana, CBD, or opioids affects pre-appointment anxiety about pain among patients seeking abortion. We hypothesized that patients with recent substance use would have more anxiety about abortion-related pain compared to patients without recent substance use.

## Materials and methods

We conducted a prospective, cross-sectional survey study of patients presenting for elective pregnancy termination procedures (dilation and curettage, medication abortion, or dilation and evacuation up to 22 weeks gestation) at an academic family planning clinic in Denver, Colorado. Our primary objectives were to better understand rates of substance use among our population and to compare worry about expected pain during or after an abortion between patients with recent use of opioid-containing substances and those without recent use of opioids. During the study period, we offered an anonymous and voluntary survey to patients ages 18-45 years, presenting to the clinic in their intake forms. The survey (Appendix) assessed both recent use (within three months) of marijuana, CBD, and opioids and use of these substances on the day of the abortion procedure. Participants rated their anxiety about pain during and after their abortion (medication or surgical) on a 0-10 scale prior to seeing a healthcare provider. The scale used both color (0=green, 10=red) and written (0= “Not Worried”, 10= “Extremely Worried”) cues to guide patients in selecting their level of worry. The survey also assessed demographic characteristics and prior abortion and obstetrical history. We placed two locked boxes (waiting room, inside clinic) where completed surveys could be placed by patients. The surveys collected no identifiable information on patients, nor were they linked to patient’s medical records. We chose to use anonymous surveys to reduce potential under-reporting of substance use by patients.

We performed all statistical analyses using SPSS™ statistical software version 27 (IBM Corp., Armonk, NY, USA). We performed descriptive analyses including tests of normality for participant demographics and characteristics. We determined median anxiety scores and used Mann-Whitney U tests to compare scores between recent substance users (marijuana, CBD, and opioid users) and non-users.

Assuming that 5% of participants would report opioid use within the last three months, we aimed to demonstrate a 2-point difference in ‘worry’ scores using our 10-point Likert scale. Assuming this scale has a 2-point standard deviation like other similar Likert scales, we would need 170 total participants (nine opioid users and 161 non-opioid users) to demonstrate our desired 2-point difference with an alpha of 0.05 and 80% power. However, since this 5% prevalence of opioid use is an estimate, we planned to include at least 200 participants.

## Results

We collected 272 anonymous surveys from November 2020 through September 2021; we excluded 55 surveys that were either incomplete or did not meet inclusion criteria. Among the remaining 217 participants, the majority were age 35 or younger (85%) and self-reported their race as White or Caucasian (50.2%) (Table 1). The majority of respondents had had previous deliveries (55%), no history of prior abortion (60.7%), and planned on a procedural or surgical abortion procedure (57%) (Table 1). For participants planning on a surgical abortion procedure, the majority planned to obtain intravenous sedation (31%) for pain control although many were undecided (28.5%). When asked about substance use within the last three months, more participants reported marijuana use (46.5%) followed by CBD (24%) or opioids (5.1%) (Table 1). Very few participants reported recent use of other substances (e.g., cocaine, methamphetamines, psilocybin mushrooms). 

**Table 1:** Demographics and reported recent substance use among patients seeking abortion services at an academic family planning clinic (N=217)

Age (years)	n (%)
18-25	70 (32.3)
26-35	114 (52.5)
36-45	33 (15.2)
Race/Ethnicity	
White	109 (50.2)
Black	23 (10.6)
American Indian	3 (1.4)
Asian	11 (5.1)
Hispanic	49 (22.6)
Mixed	7 (3.2)
Unknown/Missing	15 (6.9)
Ever had vaginal delivery or cesarean section	
Vaginal Delivery	91 (41.9)
Cesarean Section	19 (8.8)
Both	9 (4.1)
None	95 (43.8)
Missing	3 (1.4)
Ever had an abortion before	
Prior Surgical Abortion	48 (22.1)
Prior Medication Abortion	25 (11.5)
Prior Surgical and Medical Abortions	11 (5.1)
None	130 (59.9)
Missing	3 (1.4)
Plan for surgical abortion or medical abortion or unsure	
Surgical abortion	124 (57.1)
Medical abortion	63 (29.0)
Unsure	30 (13.8)
If surgical abortion is planned, what type of sedation are you planning? (n=158)	
Intravenous sedation	49 (31.0)
Oral sedation	47 (29.7)
No sedation	17 (10.8)
Unsure	45 (28.5)
Substance use in the past 3 months (reported YES)	n (%)
Opioid	11 (5.1)
Marijuana	101 (46.5)
Cannabidiol	52 (24.0)
Use the day of the appointment	
Opioid	0 (0)
Marijuana	25 (11.5)
Cannabidiol	4 (1.8)
Marijuana and Cannabidiol	10 (4.6)
Other substance use in last 3 months (reported YES)	
Cocaine	4 (1.8)
Meth	2 (0.9)
Mushrooms	4 (1.8)
Ecstasy	1 (0.5)
Acid	3 (1.4)
Other	1 (1.0)

**Figure 1:**
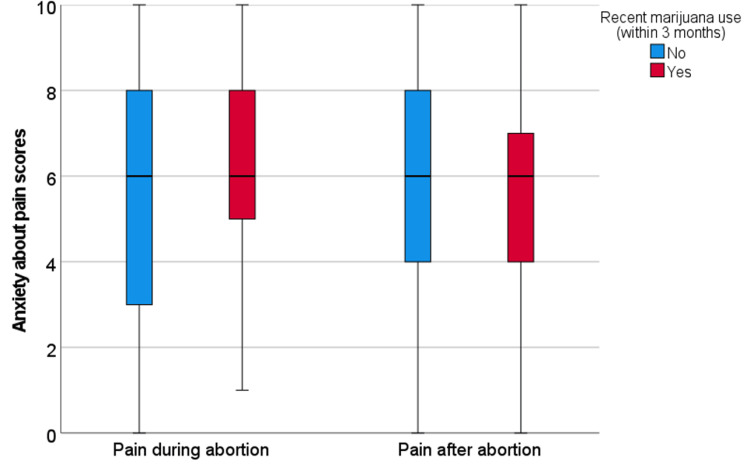
Anxiety about pain during and after abortion for participants with recent marijuana use versus all other participants Box-plots of anxiety scores for pain during and after abortion for participants who reported recent marijuana use (n=101) versus all other participants (n=116). The box represents the first and third quartiles with the band inside the box representing the median. Whiskers represent the data within 1.5 interquartile range of the upper and lower quartile.

**Figure 2:**
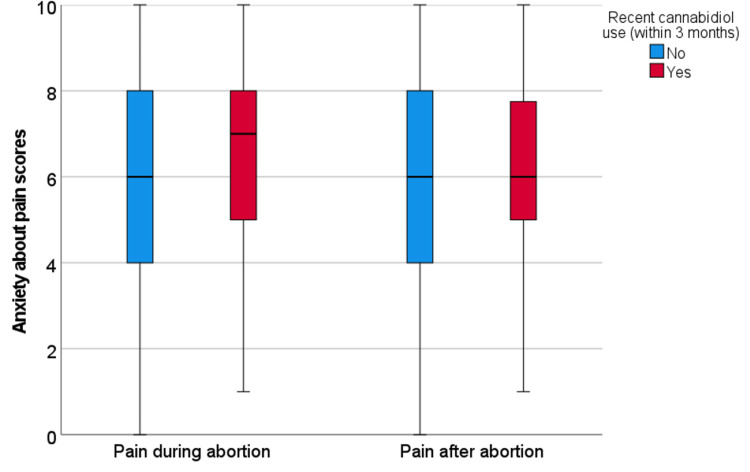
Anxiety about pain during and after abortion for participants with recent cannabidiol use versus all other participants Box-plots of anxiety scores for pain during and after abortion for participants who reported recent cannabidiol use (n=52) versus all other participants (n=165). The box represents the first and third quartiles with the band inside the box representing the median. Whiskers represent the data within 1.5 interquartile range of the upper and lower quartile.

**Figure 3:**
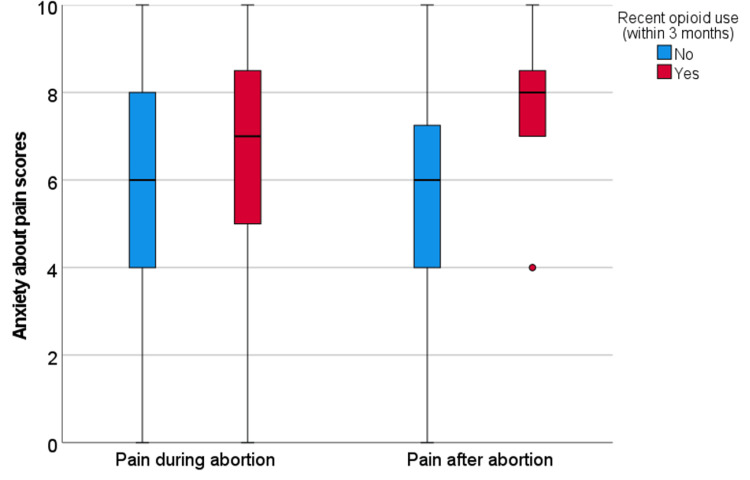
Anxiety about pain during and after abortion for participants with recent opioid use Box-plots of anxiety scores for pain during and after abortion for participants who reported recent opioid use (n=11) versus all other participants (n=206). The box represents the first and third quartiles with the band inside the box representing the median. Whiskers represent the data within 1.5 interquartile range of the upper and lower quartile. Outliers are represented by °’s (outside 1.5 times the interquartile range).

Among all participants, median anxiety scores were 6.0 (range 0-10, interquartile range 3.0) for both pain during and after abortion. Recent marijuana users reported similar anxiety scores for pain during (6.0 vs 6.0, p=0.21) and after (6.0 vs 6.0, p=0.68) to non-marijuana users (Figure 1). Recent CBD users had non-significantly higher anxiety about pain during the abortion (7.0 vs 6.0, p=0.10), but not about pain after (6.0 vs 6.0, p=0.44) compared to non-CBD users (Figure 2). For the 11 participants who reported recent opioid use, they reported higher anxiety about pain during (7.0 vs 6.0, p=0.33) and after (8.0 vs 6.0, p=0.01) the abortion as compared to non-opioid users (Figure 3). Participants who reported a history of at least one prior abortion reported lower anxiety about pain after their procedures compared to participants who reported never experiencing an abortion (5.0 versus 7.0, p=0.04).

## Discussion

We found that substance use was common in our population, with almost half of participants reporting marijuana use in the last three months, and a fourth of participants reporting CBD use. Fewer participants reported opioid use and very few participants reported use of other substances. Though opioid use was overall low in our study consistent with the national average, we found that recent opioid users had significantly greater anxiety about post-abortion pain. From prior studies, we know that anxiety, depression, and a patient's anticipation of pain are strong predictors of the pain they perceive during procedural abortion [7-11]. Many clinical, sociodemographic, and personal factors likely play a role in this anticipation of pain for abortion, but we have a paucity of literature on how recent use of substances, including opioids, can influence anxiety or worry about abortion-related pain. Our findings support that opioid use within the last three months may be a clinically important factor for increasing anxiety about abortion-related pain.

Opioid use disorder and anxiety-related symptoms co-occur at strikingly high rates [12]. Pain-related anxiety, which is worry about the negative consequences of pain, represents an area of growing interest, particularly in the field of opioid use disorder [13]. Increased levels of anxiety lead to avoidance of activities that promote pain [14]. This pattern is theorized to be cyclical, such that pain-related anxiety is linked to more severe pain and pain-related problems which further promote worry-related beliefs about the nature of pain [15]. In the setting of abortion care, patients with pain-related anxiety are facing the prospect of a procedure with at least some unavoidable aspect of pain, which could directly contribute to experienced pain levels during the abortion procedure if their pain-related anxiety is not properly addressed [6,11]. Thus, understanding substances that can have both physiologic and psychologic influences on pain perception is an important part of abortion care, particularly recent opioid use [6]. 

Although we found that recent opioid users had significantly greater anxiety about post-abortion pain, we did not see this among participants using marijuana or CBD. It is possible that since opioids greatly affect perception of pain compared to other substances, people using opioids have more pain-related concerns than people using other substances. However, this would require further investigation.

Strengths of this study include that the survey was anonymous and participants may have disclosed recent substance use more truthfully and completely than if interviewed in person or completed a survey linked to their name or medical chart. Additionally, anxiety was assessed prior to any clinical counseling, thereby avoiding potential influences of counseling on this outcome.

Limitations of this study primarily focus on the brief length and limited scope of the questionnaire used. We could not reasonably ascertain the potential reasons for opioid or other substance use, and so cannot discern recreational versus prescribed substance use. Additionally, we did not directly ask about pre-existing psychiatric diagnoses such as anxiety, which may have confounded the data. We also did not ask additional anxiety-focused questions to determine trait versus state anxiety. Finally, given the anonymous nature of the survey, we were unable to collect prospective pain data or pain data during or after abortions.

## Conclusions

Identifying recent substance use and risk factors for anxiety about abortion-related pain, such as recent opioid use can be helpful to abortion providers. This information may help to provide more individualized counseling on pain expectations, potentially leading to reduced anxiety and an improved abortion experience for this unique patient population. Future research could examine if counseling about anxiety of abortion-related pain changes patient satisfaction or abortion experience, along with pre-abortion anxiety versus actual pain scores before, during, and after abortion.

## Figures and Tables

**Figure 4:**
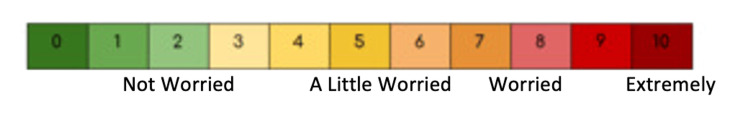
Scale

**Figure 5:**
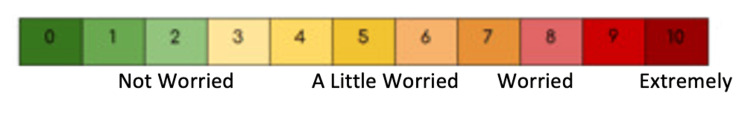
Scale

## Appendices

Thank you for filling out this survey as it will help our clinic provide better care for our patients in the future. This survey is completely voluntary and completely anonymous.  Your responses in no way will be linked to you. Your doctor will not see your responses. Your answers will not influence your medical care in any way.

1. What is your age?

□               18-25 years

□               26-35 years

□               36-45 years

□               46+ years

If you are younger than 18, you do not qualify for this study, but thank you for your interest!

2. Do you plan on having a surgical abortion or medication abortion today?

□               Surgical abortion (D&C or D&E procedure)

□               Medication abortion

□               Unsure

□               Not having an abortion today

If you answered Not having an abortion today, you do not qualify for this study, but thank you for your interest!

3. Are you planning on any additional sedation if you are having a surgical abortion/procedure? (medicine to make you comfortable/sleepy)

□               Intravenous (IV) sedation

□               Oral sedation (pills)

□               No additional sedation

□               Unsure

4.  On the following scale, please circle the number that best indicates how worried you are about the pain that you might feel DURING your surgical abortion procedure or DURING your medication abortion at home?

5. On the following scale, please circle the number that best indicates how worried you are about the pain that you might have AFTER your surgical abortion procedure or AFTER undergoing a medication abortion at home?

If you are worried about the pain you may experience during or after your abortion, please let your doctor know and we will make sure to address your concerns.

6. In the past three months, have you ever taken methadone, subutex (buprenorphine), or suboxone (buprenorphine with naloxone)? 

YES_________      NO ___________

7. In the past three months, have you taken any drugs that contain an opioid? (ex: codeine, hydrocodone, oxycodone, fentanyl, MS contin, morphine, heroin, Oxycontin, Vicodin, Percocet, Norco, Tylenol #3)

YES__________   NO___________

If you answered NO to question 7 above, please skip to question 11. Otherwise, please continue with the survey:

8. In the past 3 months, how often did you take any of these drugs?

□               Once

□               A few times (more than once but less than every week)

□               Every week

□               Every day or almost every day

9. Did you use any of these opioid-containing drugs today?

YES___________ NO___________

10.  Were you prescribed these opioid-containing drugs by a licensed healthcare provider? (doctor, dentist)

YES__________ NO____________

11. In the past three months, have you used marijuana/THC in any form? (smoke, vape, edible)

YES___________ NO_____________

12. In the past three months, have you swallowed any CBD in tincture, oil, gummy, pill, or capsule, soda forms? (e.g. CBD/cannabidiol)

YES___________ NO_____________

            If yes to 11 or 12, did you use any marijuana or marijuana-derivatives (CBD, THC) today? (Mark all that apply)

Marijuana_____    Marijuana derivatives (CBD, THC) _____    None_____ 

13. In the past three months, have you used any of the following drugs? (Mark all that apply)

□               Cocaine/crack/coke/blow

□               Methamphetamine/meth/speed

□               Ecstasy/MDMA/molly

□               Mushrooms/psilocybin mushroom/magic mushrooms/shrooms

□               PCP/angel dust

□               Ketamine/k/special k

□               Acid/LSD

□               Other:________________________________

If you have used any of these drugs, did you use it today?

YES___________ NO_____________

14. Have you ever had a vaginal delivery or cesarean delivery before?

YES___________ NO_____________

15. Have you ever had an abortion before? (Mark all that apply)

□               Yes, surgical abortion

□               Yes, medication abortion

□               No

18. Which of the following best describe you? (Mark all that apply)

□               White/Caucasian

□               Black/African American

□               American Indian/Alaskan Native

□               Asian/Pacific Islander

□               Hispanic

□               Unknown/None of the above

Thank you for completing this survey.
